# Baseline Predictors of Adherence in a Randomised Controlled Trial of a New Group Psychological Intervention for People with Recurrent Binge Eating Episodes Associated to Overweight or Obesity

**DOI:** 10.3390/nu13114171

**Published:** 2021-11-21

**Authors:** Haider Mannan, Marly Amorim Palavras, Angélica Claudino, Phillipa Hay

**Affiliations:** 1 Translational Health Research Institute, School of Medicine, Western Sydney University, Sydney 2751, Australia; marlypalavras@gmail.com (M.A.P.); p.hay@westernsydney.edu.au (P.H.); 2 Eating Disorders Program (PROATA), Department of Psychiatry, Universidade Federal de São Paulo (UNIFESP), São Paulo 04017030, Brazil; amclaudino@gmail.com

**Keywords:** binge eating disorder, bulimia nervosa, cognitive therapy, obesity, patient dropout

## Abstract

Purpose: Understanding the high rate of treatment adherence in trials of people with eating disorders is important as it can compromise the quality of the trials. In clinical practice, it may also contribute to illness chronicity, relapse, and costs. Thus, we investigated factors associated with adherence to a new treatment HAPIFED, which integrates cognitive behavioural therapy having extended sessions with body weight loss therapy compared to cognitive behavioural therapy with extended sessions alone, for individuals with Bulimia Nervosa or Binge Eating Disorder or other eating disorders comorbid with overweight or obesity. Methods: In total, 98 participants having bulimia nervosa, binge eating disorder and other specified and unspecified eating disorders were recruited with 50 randomised to HAPIFED and 48 to the control intervention CBT-E, all administered in groups of up to 10 participants. An investigator external to the site conducted the random allocation, which was concealed from the statistician involved in the analysis, and known only to the therapists until the finalization of the 12-month follow-up after the end of active treatment. Three scenarios in the timeline treatment of a total of 30 sessions were assessed: 33% or 60% or 75% of presence. Mixed-effects logistic regression analysis was performed to find the correlates of adherence after adjusting for clustering by number of group participants. To account for heterogeneity by types of eating disorders in the sample, the latter variable was considered as a control factor in the models. A subgroup analysis was performed for those with binge eating disorder as this was the largest (N = 66) eating disorder group. Results: None of the six variables—frequency of binge eating episodes, purging, eating disorder symptom severity, weight, illness duration and mental health-related quality of life—significantly predicted adherence at 33%, but longer illness duration predicted higher treatment adherence at both 60% and 75% presence of the interventions. Also for 75% presence, higher body weight predicted lower treatment adherence. For the subgroup analysis, those having higher illness duration had significantly higher odds of treatment adherence for 60% and 75% of the sessions. Conclusions: Higher adherence due to late treatment completion was associated with longer binge eating illness length and a lower body weight. More research is needed to recognize factors that may interfere with engagement in treatments aiming to avoid early dropout.

## 1. Background

A challenge in the care of people with eating disorders is the high rate of attrition of treatments, varying between 29% and 73% in outpatient studies [1]. Fassino et al. [1] argued that such a high dropout can compromise the quality of the trials, reducing power and increasing the likelihood of Type I and II errors. In clinical practice, it may also contribute to illness chronicity, relapse, and costs [1]. A recent systematic review, evaluated predictors in following manualised CBT, among samples with BN, Binge Eating Disorder (BED) and mixed samples including normal weight eating disorders [2]. Similar results for sociodemographic aspects and eating disorder severity were found in a comprehensive review, but the authors noted that general psychopathology favours the non-sequence of treatment, and that binge-purging subtype of anorexia nervosa, two borderline personality disorder traits (high maturity fear and impulsivity) and two psychological traits (high maturity fear and impulsivity) are predictors for dropouts in eating disorders treatment [1]. In another systematic review and meta-analyses [3], weight suppression, more frequent binge eating and purging behaviours, less motivation for the treatment and avoidant attachment associated with binge/purge subtype were more likely to predict dropout. There are many factors that relate to treatment engagement, factors in the treatment alliance and type of therapy offered [4]. This present paper examines the pre-treatment characteristics of participants, knowledge of which may aid clinicians in tailoring or targeting therapy efforts cognizant that people with these characteristics may be more likely to disengage with the therapy. It is also clear that more studies need to be undertaken to determine risk factors for attrition or treatment outcome in internet-based interventions for BED [5]. Jensen et al. [5] found minor differences between completers and non-completers on depression and no differences in BED-symptoms, BMI, and sociodemographic variables. Participants who completed treatment showed large reductions in eating disorder pathology.

A single-blinded RCT evaluated the effectiveness of a manualised psychotherapy for people with BN or BED comorbid with high body mass index (BMI) compared to the CBT-E [6]. The main aspect of this new intervention named HAPIFED is the integration of features of CBT-E and BWLT with the purpose of promoting weight loss and binge eating remission. We investigated the efficacy and safety of introducing a weight loss intervention to the treatment of people with disorders of recurrent binge eating and a high body mass index.

To our knowledge, there has been no previous trial of an integrated CBT-E with BWLT for disorders of binge eating in people with high BMI. A special feature of HAPIFED is a higher number of sessions when compared to CBT-E and other behavioural weight loss programs commonly offered. The reason for extending the treatments in this trial to over six months was to provide sufficient time for implementing the cognitive work as well as the necessary behavioural changes. To avoid bias related to treatment intensity and duration, CBT-E broad version was used, which was extended to 30 sessions incorporating additional modules addressing psychological-maintaining factors, namely, clinical perfectionism, core low self-esteem and interpersonal problems. All participants were evaluated in three stages comprising 22 out of 30 sessions; so, it was a meaningful extension of CBT-E.

In the main trial for HAPIFED [6], 100 Brazilian participants aged ≥ 18 years, with a diagnosis of bulimia nervosa or binge eating disorder, BMI > 27 to <40 kg/m^2^ were recruited from both community and clinics and individually randomised to a therapy arm. The sample size 100 was based on a priori power analysis that used an estimate of a moderate between group effect size (i.e., 0.6) in the primary outcome of weight loss. To achieve this with power of 0.8 and 1-tailed alpha 0.05, a minimum of 36 participants per group was required, according to Cohen’s tables. Allowing for attrition, the sample size was 50 per arm and 10 per therapy group. In the trial, participants were randomly offered 30 sessions of one of the interventions in a group format. Five groups of ten participants received the experimental intervention (HAPIFED) and the other five groups of ten the control intervention (CBT-E). Both therapies were manualised, and the RCT comprised 1 individual session and 29 office-based group sessions over 6 months. Assessment points were at baseline, end of therapy, and 6 and 12 months after end of therapy. The primary outcome of the intervention was reduced weight. Secondary outcomes were improved metabolic indicators of weight management, reduction in eating disorder symptoms including improved control over eating, improved adaptive function, physical and mental health-related quality of life, and reduced levels of depression and anxiety. The results [7] showed that HAPIFED was not superior to CBT-E in promoting clinically significant weight loss and was not significantly different in reducing most ED symptoms. No harm was observed with HAPIFED, in that no worsening of ED symptoms was observed.

The aim of this study is to evaluate features that can predict adherence to psychological treatments (combined or not to multidisciplinary interventions as in HAPIFED) for individuals with BN or BED associated with high BMI. This study will investigate the results of the active phase of treatments only (blinded investigation). Three points in the timeline of the 30 sessions that are considered for examination are 33%, 60% and 75% of treatment sessions completed (the rationale is discussed below in the first sub-section under Methods). We use mixed-effects logistic regression to determine the correlates of eating disorder treatment completers versus non-completers which is a dichotomous variable.

As exploratory hypotheses, we anticipate that frequency of binge eating episodes, presence of purging behaviour, and eating disorder symptom severity would be associated with drop out. Further, we investigate the following putative predictors of dropout: mental health-related quality of life, illness duration and weight. The term “drop out” is used to describe both the unilateral ending of regular treatment by a patient and the decision for administrative discharge made by a treatment team.

## 2. Materials and Methods

### 2.1. Definitions

1. For the purpose of this study, we considered three scenarios in the timeline treatment of a total of 30 sessions (1 individual + 29 group sessions). The aim was not to investigate “percentage of sessions attended” but rather the timing of attrition (early/middle/late). So, we present 3 time points in a way that there is a continuum:
  - 33% of presence, corresponding to 10 sessions with no other requirement.
  - 60% of presence, corresponding to 18 sessions with no other requirement.
  - 75% of presence, i.e., (i) completed the treatment until the final session and missed less than 4 sessions in a sequence, and/or (ii) completed at least 22 out of 30 sessions.

The 33% treatment completion is clinically relevant, as it is of interest who will engage in treatment. Early dropouts are of concern as they benefit little from the intervention. The 75% treatment completion is also clinically relevant as it reflects those who will complete most of treatment, and thus may benefit most from the intervention. So, both 33% and 75% treatment completions have clinical utilities. However, we also chose 60% treatment completion on the ground that there is no consensus on what constitutes treatment completion or the number of sessions for treatment to be completed [1,8]. However, late attrition is usually 75% or more of therapy sessions and has been applied in previous studies of the authors [8].

2 Frequency of binge eating episodes was considered when the participant reported at least one episode per week during the last 3 months.
3 Purging was defined as any current use of self-induced vomiting, laxatives, and/or diuretics as a method of weight and/or shape control within the past 3 months. It could be compensatory or non-compensatory.

### 2.2. Participants

We aimed to include 100 participants in the protocol, but had to stop at 98 people, when two participants were randomised but failed to engage in treatment due to time to finish the trial allowed by the research funding agency. Thus, this secondary analysis study comprised a sample of 98 participants, adults, both genders, with BMI ≥ 27 and <40 kg/m² (either overweight or obese), recruited from clinical and community sources, with threshold or subthreshold BN or BED diagnoses, according to the Diagnostic and Statistical Manual of Mental Disorders, fifth edition (DSM-5) [9].

Sixty-six (67.3%) participants met the DSM-5 diagnostic criteria for BED and 13 (13.3%) for BN. Of the remaining 19 participants, 5 (5.1%) had OSFED BED-type, 7 (7.1%) had OSFED BN type and 7 (7.1%) UFED. Of those receiving UFED diagnosis, all reported regular recurrent binge eating, but five did not fulfil Criterion B or C for BED; one with recurrent binge eating and self-induced vomiting episodes did not meet Criterion D for BN, and; one met all criteria for BN, but only reported subjective binge eating.

The group with threshold or subthreshold BN or BED diagnoses is not a very heterogeneous group as there are some overlaps between the two conditions. We were interested in studying effects of HAPIFED on eating disorders symptoms and weight, so we expected that cases with related features would benefit from treatment. Additionally, the observation that it is common to include subthreshold cases in RCTs has led proposals for revisions of classificatory systems to widen the diagnosis of the main categories [10]. The condition BN includes recurrent binge-eating episodes or the consumption of abnormally large amounts of food in a short period of time which is followed by self-induced vomiting, strict dieting, over-exercising and/or the misuse of laxatives, enemas or diuretics. On the contrary, if one consistently eats large amounts of food, and those eating episodes cause shame, regret, guilt, or sadness, one may have BED.

### 2.3. Exclusion Criteria

The exclusion criteria were as follows: use of weight loss medication; clinical conditions that could interfere with appetite regulation; history of bariatric surgery; current diagnosis of psychosis or bipolar disorder; high level of suicide risk; current participation in psychotherapy for eating disorders. This RCT was conducted by specialists at a university centre for treatment of eating disorders (PROATA) in the Universidade Federal de São Paulo, Brazil. Participants were recruited from July 2015 to November 2017, via waiting list, advertisements on the internet, printed and oral media.

### 2.4. Stages of Evaluation

**Figure 1:**
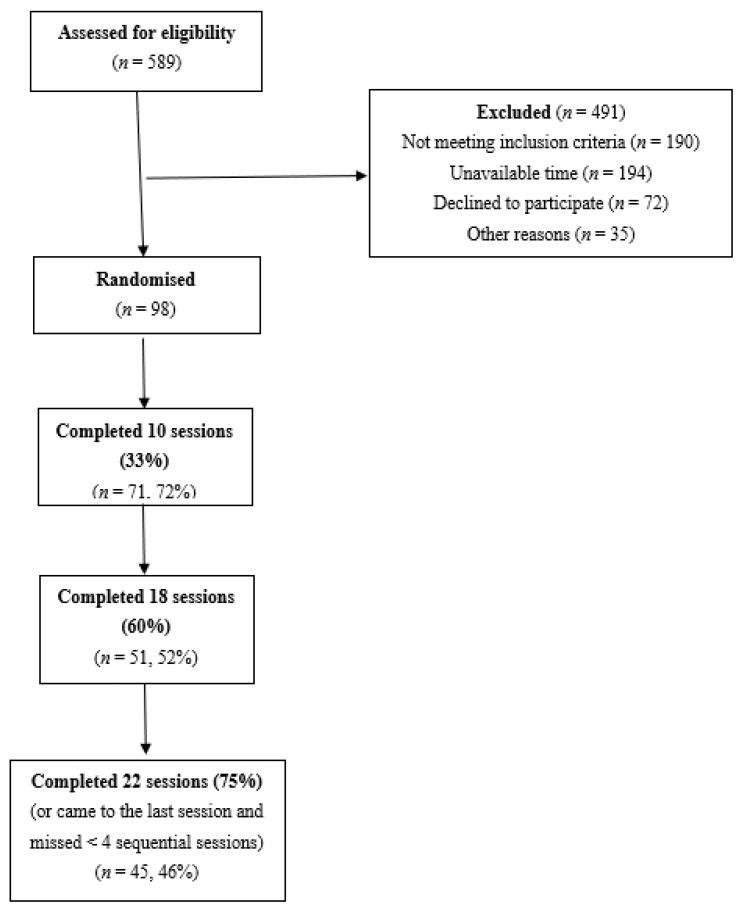
Flow chart diagram.

All participants were evaluated in three stages (see trial protocol for details) [4]. The third stage comprised the assessment with a semi-structured interview that confirmed the eating disorder diagnosis and detailed eating disorders symptoms and behaviour. In total, 10 groups were organised, with five groups receiving the experimental intervention (HAPIFED) and the other five groups receiving the control intervention (CBT-E). An investigator (PH) external to the site conducted the allocation through a website www.sealedenvelope.com (accessed on 2 September 2015). The randomisation process was concealed from the statistician involved in the analysis, and only the therapists of the Brazilian research team knew it until the finalization of the 12-month follow-up after the end of the active treatment. See the flow chart (Figure 1).

Four female therapists were guided in agreement with CBT-E and HAPIFED manuals. They were trained by experienced therapists (PH and JS) receiving monthly telephone supervision during the two pilot groups, and monthly telephone supervision during the trial by PH who visited Brazil six times in 3 years for face-to-face supervisions with the Brazilian therapists and other members of the HAPIFED project. Each pair of therapists conducted both CBT and HAPIFED groups to administer the non-specific therapists’ effect.

Both interventions offered an initial individual session and a further 29-group sessions, being twice weekly for the first four weeks and weekly after that until the end of active treatment comprising a total of 6 months. HAPIFED is a multidisciplinary program including four sessions with dietician and/or occupational therapist accompanied by the psychological therapists. Differently from CBT-E, HAPIFED emphasizes a nutritional counselling given by the nutritionist, the behavioural monitoring including appetite cues directed to the hunger and satiety perception, the behavioural activation, e.g., stimulate the remission of body avoidance, healthy exercise encouragement, and emotion regulation skills mainly focusing on mood intolerance. Despite CBT-E being originally offered in 20 sessions, in this protocol the number of sessions were extended in number of sessions and duration to equate to HAPIFED. Additionally, both interventions received four group follow-up sessions during the first 6 months’ follow-up after the end of the active treatment, and a final assessment was conducted in 12-month follow-up.

### 2.5. Measures

For the purposes of this study, the following instruments were used:

1. For the evaluation of frequency of binge eating episodes, purging behaviour and eating disorder symptom severity: the semi-structured EDE Edition 17.0D [11] interview was used. The EDE generates eating disorder diagnoses and assesses the symptom severity using four subscales, which are averaged for a global score. The version 16.0 was translated to Brazilian/Portuguese with a satisfactory reliability (80% inter-interviewer agreement and 0.69 Kappa were evaluated with considering the diagnosis using the EDE interview) and concurrent validity (77.3% agreement and 0.68 kappa). For a consistency with the most recent edition—EDE 17.0D—small modifications were made in the previous Portuguese version, in order to derive DSM-5 diagnoses.
2. After a first screening by telephone, the eligible participants were invited for a first presencial interview when the informed written consent was signed, the inclusion and exclusion criteria were rechecked, and a medical history and physical examination were conducted by a clinical physician. Weight and height were measured using a calibrated, electronic digital scale and a stadiometer, from which BMI (kg/m²) was calculated.
3. For the evaluation of mental health-related quality of life: the SF-12 [12] was applied. The SF-12 is a self-report instrument that measures physical and mental health-related quality of life.
4. For the measurement of eating disorder illness duration: the participants fulfilled a self-report questionnaire in which they were asked about the illness duration. In the first interview, a self-reported questionnaire was completed by the participants with sociodemographic information (age, sex, occupation, marital status, etc) and the illness duration where they needed to complete a space with years and months, e.g., Illness duration: …… years and …… months.

### 2.6. Participant Flow

As we aimed to study early, middle and late treatment attrition three time points, 33%, 60% and 75%, were analysed. Figure 1 is a flowchart showing how the eligible number (*n* = 98) of participants was arrived at and the number of participants completing at least 10 or 33% sessions, at least 18 or 60% sessions and at least 22 or 75% sessions. This figure shows that the number of participants for these treatment attrition three time points were 71, 51 and 45, respectively. 

### 2.7. Statistical Analyses

Data were cleaned including correcting for coding errors. Descriptive statistics such as mean for a continuous covariate and its standard error, as well as proportion for a categorical covariate and its standard deviation were estimated for completers and non-completers of sessions. It was clinically more useful to know if sufficient sessions were attended so that a therapist would be satisfied the person had ‘completed’ therapy. Fewer sessions than this indicated the therapy may have been less effective because it was incomplete. So, the dependent variable identified a decision issue and hence was clearly dichotomous and so treating it as continuous would not be informative to clinicians. Logistic regression analysis was thus performed to determine the significant predictors of treatment completion. A random intercept model under the mixed effects framework was used to account for adjustment of clustering by number of group sessions attended. To reduce or suppress the effect of heterogeneity in the sample due to a mixture of different eating disorder types, we included in the models a categorical variable indicating eating disorder types. To fit the logistic models for predicting the odds of completing at least 18 and 22 sessions, we used the multilevel adaptive Gauss–Hermite quadrature approximation of maximum likelihood for rare events as these models did not satisfy the widely used criterion of having at least 10 events per predictor for using maximum likelihood in logistic models. This method is the most accurate in estimating unbiased regression coefficients in such situations [13]. The missing data were estimated in the analysis by multiple imputation using multivariate normal imputation. All analyses were performed using SAS version 9.4 [14]. SAS proc glimmix method = quad (fastquad qpoints = 3) sub-option was used to perform the analysis for rare events. In total, three mixed effects logistic regression models were fitted for both the whole sample and the BED subgroup when the dependent variables were:

(i) Completion of 33% or 10 sessions,
(ii) Completion of 60% or 18 sessions, and
(iii) Completion of 75% or 22 sessions.

## 3. Results

A total sample of 98 participants were included in this study of which 50 were randomly allocated to the treatment group and the remaining 48 to the control group. Out of 98 participants, 66 (67.3%) participants met the DSM-5 diagnostic criteria for BED, 5 for OSFED BED- type, 5 for regular BE. Of the remaining participants, 13 (13.3%) and 9 had BN (threshold) and recurrent BN (OSFED), respectively. The majority were women (*n* = 94, 96%), Caucasian (*n* = 73, 74.5%), and were employed (*n*= 59, 60.2%). Forty-five percent were married (*n* = 44) and 43% (*n* = 42) completed tertiary education. The mean age was 40.55 years (SE 1.18), mean weight measured at baseline was 89.26 kg (SE 1.27), mean BMI at baseline was 33.68 (SE 0.33), and the baseline weight range was 61.9–114.8.

For the whole sample, the mean values for illness duration, eating disorder symptom severity score, mental health-related quality of life, binge eating frequency and baseline weight were 14.98 (SE 1.15), 2.56 (SE 0.08), 35.59 (SE 1.10), 39.40 (SE 3.00), 88.27 kg (SE 1.27), while 18.37% (SE 0.039) performed purging. These results are presented in Table 1.

For those with binge eating disorder (*n* = 66), the mean values for illness duration, eating disorder symptom severity score, mental health-related quality of life, binge eating frequency and baseline weight were 14.48 (SE 1.43), 2.44 (SE 0.09), 35.08 (SE 1.47), 38.56 (SE 3.23), 88.11 kg (SE 1.61) while 3.03% (SE 0.02) performed purging. These results are presented in Table 2.

Six baseline (pre-randomisation) features with a putative association with treatment completion were analysed—frequency of binge eating episodes, presence of purging behaviour, eating disorder symptom severity, mental health-related quality of life, illness duration and baseline weight. No significant statistical differences were found for these variables between participants who completed 33% of the sessions. For those who completed 60% session, there was a significant difference for illness duration. Those having higher illness duration had significantly higher odds of treatment completion. No significant differences for frequency of binge eating episodes, purging behaviour, eating disorder symptom severity and mental health-related quality of life were found between participants who had 33%, 60% and 75% or the most stringent of treatment completions, respectively. Participants who achieved the most stringent treatment completion criterion had significantly lower baseline weight (*p* = 0.04) and longer illness duration (*p* = 0.01) than those who did not complete 75% of sessions. (See Table 3).

For those with binge eating disorder (*n* = 66), illness duration was the only significant variable between participants who completed 60% and 75% of the sessions. Similar to the results obtained for the whole sample, those having higher illness duration had significantly higher odds of treatment completions for 60% and 75% of the sessions. (See Table 4).

## 4. Discussion

This study investigated putative predictor variables of treatment completion in a sample of 98 participants of a RCT testing the efficacy of a multidisciplinary intervention (HAPIFED) for people with BN or BED comorbid with high BMI against a control therapy (CBT-E). Six potential predictor variables measured at baseline were considered in this study—frequency of binge eating episodes, presence of purging behaviour, eating disorder symptom severity, mental health-related quality of life, illness duration and weight—and their impact in three periods of treatment completion were analyzed. The treatment group was excluded as a predictor because this present paper examines the pre-treatment characteristics of participants, whose knowledge may aid clinicians in tailoring or targeting therapy efforts, while being aware that people with these characteristics may be more likely to disengage with the therapy. Additionally, treatment group is unlikely to be an independent predictor in our models because the participants were not aware of which group they were in, and thereby could not have favoured one over the other. This is because in this study participants were blinded to groups. To assess the effectiveness of blinding we tested whether there were significant differences in completion of 10, 18 or 22 sessions between the two groups and found that there was not any (*p* > 0.05). So, the blinding process in no way influenced adherence to treatment. Also, treatment group is likely to confound with some of the predictors already included in the models, such as duration of binge eating illness, binge eating frequency, binge eating symptom severity, purging and with body weight probably to a lesser extent [7]. Similarly, the other predictors included in the models were within the aims of our study in terms of chosen predictors as stated above.

Purging was included as a predictor because it is one of the most consistent predictors of outcome in the literature. Also, people with BN do not always purge because there are two types, purging BN and non-purging BN. People with non-purging type of BN fast or perform compulsive exercise. It differs from BED by the fact that people with non-purging type BN have regular extreme weight control behaviours while people with BED do not. People with BED can also purge similar to those with purging BN, but they do it less frequently (less than weekly). Although the DSM-5 [9] states “the binge eating is not associated with the recurrent use of inappropriate compensatory behaviour as in bulimia nervosa”, it is often assumed to infer total absence of these behaviours frequently as well as ‘absence of recurrent behaviours’, i.e., they may have the behaviours but only infrequently and not recurrently.

There were also some participants in our study having non-purging type of BN, e.g., in our sample, out of 20 people with BN (13 threshold, seven subthreshold), four were non-purging. In the regression analysis we did not focus on differential diagnosis because it is likely to be confounded with binge eating frequency, binge eating duration, binge eating symptom severity, purging etc., which were included as predictors in the models. SF-12 was used because it is an indicator of mental health related quality of life, while the primary aim of HAPIFED was.to improve overall health and wellbeing, rather than just reduction of body weight. The latter was only a secondary aim, because of which, SF-12 was considered as a predictor of attrition in an RCT to investigate the effect of HAPIFED in comparison to BWLT.

None of the six variables investigated significantly predicted adherence at 10 sessions (33%) of the interventions. For the period of 18 sessions (60%), longer illness duration predicted higher treatment adherence while for 22 sessions (75%) both lower weight and longer illness duration predicted higher treatment adherence.

Some studies investigated the impact of illness duration and weight on outcomes in treatment, but not on prediction of treatment completion [2,3]. In the present study, adherence decreased over the course of therapy, and particularly after 33% of sessions. The duration of the intervention in this RCT was longer than the usually reported—six months of active treatment—what may have affected the increasing dropout rates along the treatment. Another possible explanation for the non-completion of treatments in our study may be related to difficulties in dealing with the cognitive and behavioural changes proposed in therapies within a sample of individuals with recurrent binge eating associated with high BMI.

Nackers et al. [15] highlighted the importance of addressing the commonly observed individuals’ desire to lose a high proportion of weight in a short period of time.

We hypothesised that, in our study, those with lower baseline weight were more motivated to accept the increased focus of treatments on the critical examination of unrealistic expectations of achieving an “ideal weight” versus “real weight” and on the perception of the internal cues that regulate appetite regulation (HAPIFED).

It is possible that a longer eating disorder illness or chronicity may be associated with poorer health and less motivation to change and stay in therapy—at least up to 60% of sessions. Furthermore, this effect may not reduce over time as it was found at 75% of sessions that a longer duration was still positively associated with treatment completion.

Strengths of this study included using an RCT having longer duration of the intervention than usual as it allowed examination of attrition and its correlates over three time points. When number of events (adherence) per predictor was inadequate to perform maximum likelihood estimation using logistic regression, the adaptive Gauss–Hermite approximation of maximum likelihood was applied. Limitations of this study included the inability to investigate associations between adherence and substance use or personality disorder, as these were too infrequent in the sample. Other limitations include having only four men out of 98 participants, thus limiting generalizability and precluding subgroup analysis. It may be harder to predict earlier adherence compared to later adherence. The heterogeneous sample and the different treatments may have contributed to the difficulty in predicting adherence. Due to using a heterogeneous sample, the severity of ED symptoms was likely to have differed between participants, so we included the severity of ED symptoms as one of the predictors of adherence the treatments. We suggest using a less heterogeneous and larger sample for a future study. The limitation of our subgroup analysis for those with binge eating disorder is that the patients were no longer randomly distributed between the treatment and control groups; additionally, the sample size was only 66 participants.

## 5. Conclusions

Similar to any study assessing the effect of an intervention on outcome, low adherence may also impair the quality of studies evaluating interventions for disorders of recurrent binge eating associated with high BMI. However, predictors of treatment completion have not been consistently established. This study found that a longer binge eating illness duration and lower baseline body weight predicted completion of 75% of treatment sessions, and longer binge eating illness duration predicted completion of 60% of sessions of psychological interventions for these disorders. More research is needed to recognize factors that may interfere with engagement in treatments, aiming to avoid early dropout, relapses and chronicity.

## Figures and Tables

**Table 1:** The mean/percentage distribution of the predictors in the whole sample.

Predictors.	Whole Sample (*n* = 98)	
	Mean/Percentage	SE
MHRQoL	35.59	1.10
EDE global score	2.56	0.08
Weight/kg	88.27	1.27
Illness duration	14.98	1.15
Binge eating frequency	39.40	3.00
Purging behaviour	18.37%	0.04

**Table 2:** The mean/percentage distribution of the predictors among those with BED.

Predictors	Whole Sample (*n* = 66)	
	Mean/Percentage	SE
MHRQoL	35.08	1.47
EDE global score	2.44	0.09
Weight/kg	88.11	1.61
Illness duration	15.48	1.43
Binge eating frequency	38.56	3.23
Purging behaviour	3.03%	0.02

**Table 3:** Results of descriptive statistics at baseline and logistic regression for presence of 33% sessions, 60% sessions and 75% sessions, respectively.

		33% Sessions of Presence				60% Sessions of Presence				75% Sessions of Presence		
Baseline Feature (Type of Variable)	Descriptive statistics N Mean (SE)		Logistic regression Odds Regression Ratio Coefficient (*p* value)		Descriptive statistics N Mean (SE)		Logistic regression Odds Regression Ratio coefficient (*p* value)		Descriptive statistics N	Mean (SE)	Logistic regression Odds Regression Ratio Coefficient (*p* Value)	
MHRQoL Completers	71	36.31 (1.33)	1.02	0.016	51	36.14 (1.56)	1.00	0.007	45	36.38 (1.58)	1.02	0.018
Non-completers (continuous)	27	33.85 (1.97)		(0.55)	47	34.98 (1.58)		(0.76)	53	35.00 (1.46)		(0.44)
EDE global score Completers	71	2.52 (0.10)	0.95	−0.048	51	2.57 (0.12)	1.15	0.139	45	2.64 (0.13)	1.66	0.507
Non-completers (continuous)	27	2.68 (0.13)		(0.89)	47	2.56 (0.12)		(0.68)	53	2.49 (0.11)		(0.16)
Weight/kg Completers	71	87.98 (1.44)	0.97	−0.026	51	86.97 (1.65)	0.97	−0.032	45	86.20 (1.71)	0.96 *	−0.038
Non-completers (continuous)	27	92.65 (2.57)		(0.13)	47	91.77 (1.91)		(0.10)	53	91.87 (1.79)		(0.04)
Illness duration Completers	71	15.86 (1.26)	1.02	0.02	51	17.42 (1.39)	1.05 *	0.048	45	18.29 (1.52)	1.07 *	0.068
Non-completers (continuous)	27	13.66 (2.22)		(0.46)	47	12.09 (1.61)		(0.04)	53	12.69 (1.49)		(0.01)
Binge eating frequency Completers	71	41.38 (3.84)	1.01	0.012	51	40.76 (4.09)	1.00	0.002	45	42.62 (4.50)	1.00	0.003
Non-completers (continuous)	27	33.89 (4.07)		(0.29)	47	37.91 (4.44)		(0.80)	53	36.51 (4.04)		(0.69)
		Percent (SE)				Percent (SE)				Percent (SE)		
Purging behaviour Completers	71	15.5 (0.04)	1.84	0.611	51	0.16 (0.05)	1.51	0.409	45	17.78 (0.06)	4.17	1.428
Non-completers (dichotomous)	27	18.5 (0.08)		(0.565)	47	0.21 (0.06)		(0.668)	53	15.10 (0.05)		(0.18)

Note: * indicates *p* ≤ 0.05; odds ratios and regression coefficients are all adjusted for Bulimia Nervosa vs. Binge Eating Disorder and other Eating Disorders vs. Binge Eating Disorder as controls. EDE = Eating Disorder Examination; MHRQoL = Mental Health-Related Quality of Life. In the logistic regression models, for purging, the reference category is ‘non-purging’. The *p* values for regression coefficients are based on Student’s *t* test.

**Table 4:** Results of descriptive statistics at baseline and logistic regression for presence of 33% sessions, 60% sessions and 75% sessions, respectively among those with BED.

		33% Sessions of Presence				60% Sessions of Presence				75% Sessions of Presence		
Baseline Feature (Type of Variable)	Descriptive statistics *N*	Mean (SE)	Logistic regression Odds Ratio	Regression Coefficient (*p* Value)	*N*	Descriptive statistics Mean (SE)	Logistic regression Odds Ratio	Regression Coefficient (*p* Value)	*N*	Descriptive statistics Mean (SE)	Logistic regression Odds Ratio	Regression Coefficient (*p* Value)
MHRQoL Completers	48	34.20 (1.62)	0.99	−0.001	37	34.74 (2.01)	0.99	−0.01	47	34.30 (1.72)	1.00	0.001
Non-completers (continuous)	18	34.65 (2.36)		(0.98)	29	35.50 (2.18)		(0.64)	19	35.00 (1.46)		(0.98)
EDE global score Completers	48	2.21 (0.21)	0.79	−0.24	37	2.46 (0.13)	1.09	0.09	47	2.64 (0.13)	0.58	0.26
Non-completers (continuous)	18	3.02 (0.25)		(0.59)	29	2.42 (0.13)		(0.84)	19	2.39 (0.11)		(0.54)
Weight/kg Completers	48	85.18 (1.04)	0.99	−0.012	37	86.25 (1.99)	0.97	−0.03	47	86.86 (1.90)	0.86	−0.03
Non-completers (continuous)	18	94.24 (2.88)		(0.62)	29	90.47 (2.62)		(0.23)	19	91.87 (1.79)		(0.15)
Illness duration Completers	48	14.18 (1.34)	1.01	0.012	37	18.22 (1.76)	1.07 *	0.07	47	17.02 (1.63)	1.07 *	0.07
Non-completers (continuous)	18	11.66 (2.02)		(0.70)	29	11.91 (2.26)		(0.03)	19	12.69 (1.49)		(0.01)
Binge eating frequency Completers	48	38.38 (3.14)	1.00	0.002	37	42.03 (5.19)	1.01	0.01	47	38.87 (4.23)	1.01	0.014
Non-completers (continuous)	18	35.19 (4.36)		(0.86)	29	34.14 (3.12)		(0.38)	19	36.51 (4.04)		(0.29)

Note: * indicates *p* ≤ 0.05. EDE = Eating Disorder Examination; MHRQoL = Mental Health-Related Quality of Life. Purging is excluded as a predictor because there were no purgers among those having BED. The *p* values for regression coefficients are based on Student’s *t* test.

## Data Availability

The datasets generated during and/or analysed during the current study are available from the corresponding author on reasonable request.

## Abbreviations

CBT	Cognitive Behavioural Therapy
BN	Bulimia Nervosa
BED	Binge Eating Disorder
BMI	Body Mass Index
RCT	randomised controlled trial
CBT-E	Cognitive Behavioural Therapy—Enhanced
HAPIFED	Healthy Approach to Weight Management and Food in Eating Disorders
BWLT	Behavioural Weight Loss Therapy
ED	Eating Disorder
DSM-V	Diagnostic and Statistical Manual of Mental Health 5th Edition
OSFED	Other specified feeding or eating disorder
UFED	Unspecified feeding or eating disorder
PH	Phillipa Hay
JS	Jessica Swinbourne
EDE	Eating Disorder Examination
SF12	12-Item Short Form Survey
BE	Binge Eating
SE	Standard Error
